# Carcinoembryonic Antigen Increase in a Patient with Colon Cancer Who Have Achieved Complete Remission and Negative ^18^F-FDG PET/CT: Don’t Forget the Thyroid!

**DOI:** 10.3390/curroncol28040261

**Published:** 2021-08-05

**Authors:** Alexandre Lugat, Pauline Hulo, Catherine Ansquer, Yann Touchefeu, Eric Mirallié, Jaafar Bennouna, Delphine Drui

**Affiliations:** 1 Department of Endocrinology, l’Institut du Thorax, CHU de Nantes, Hôpital Nord, 44000 Nantes, France; delphine.drui@chu-nantes.fr; 2 Center for Research in Cancerology and Immunology Nantes-Angers (CRCINA), University of Nantes, INSERM UMR 1232, 44000 Nantes, France; pauline.hulo@chu-nantes.fr (P.H.); catherine.ansquer@chu-nantes.fr (C.A.); yann.touchefeu@chu-nantes.fr (Y.T.); jaafar.bennouna@chu-nantes.fr (J.B.); 3 Thoracic Oncology Unit, CHU de Nantes, Hôpital Nord, 44000 Nantes, France; 4 Nuclear Medicine Department, CHU de Nantes, Hôtel-Dieu, 44000 Nantes, France; 5 Digestive Oncology, Institut des Maladies de l’Appareil Digestif, CHU de Nantes, Hôtel-Dieu, 44000 Nantes, France; 6 Department of Digestive and Endocrine Surgery, Institut des Maladies de l’Appareil Digestif, CHU de Nantes, Hôtel-Dieu, 44000 Nantes, France; eric.mirallie@chu-nantes.fr

**Keywords:** carcinoembryonic antigen, medullary thyroid carcinoma, colon adenocarcinoma, calcitonin

## Abstract

Serum carcinoembryonic antigen (CEA) is a tumor marker especially used to follow a patient with colorectal cancer. However, it is non-specific and could be increased in several cancers and some benign conditions. We report the case of a 70-year-old man followed since 2014 for a left colon adenocarcinoma with the persistence of an increased CEA. There was no evidence of recurrence, but a right lobar thyroid nodule without a significantly increased uptake was incidentally discovered on the CT scan of ^18^F-fluorodeoxyglucose (^18^F-FDG) PET/CT. We suspected a medullary thyroid carcinoma (MTC) explaining the persistent elevation of CEA. Plasma calcitonin levels were 47 ng/L (N < 10). Fine needle aspiration cytology found atypia of undetermined significance and the patient was reluctant to undergo surgery without any further exploration. We performed a ^18^F-fluorodihydroxyphenylalanine (^18^F-FDOPA) PET/CT preoperatively which revealed a punctiform focus of the right thyroid lobe corresponding to a pT1aN1aMxR0 medullary thyroid carcinoma, histopathologically confirmed. This case highlights that despite the potential usefulness of ^18^F-FDG PET/CT in case of an unknown source of elevated CEA this imaging may be falsely negative as in the case of MTC and should lead to further explorations.

## 1. Introduction

Serum carcinoembryonic antigen (CEA) is a member of a family of cell surface glycoproteins involved in cell adhesion [1] that is used as a tumor marker. The interest in monitoring CEA post-operatively in colorectal cancer (CRC) was shown in several studies, and it is related to an improved overall survival [2,3]. International guidelines recommend performing CEA testing every three to six months, for at least three years then every 6–12 months at years four and five after initial surgery of a localized CRC [4], and every two to three months in case of metastatic CRC [5].

However, CEA is not specific to CRC and can be increased in several cancers such as pancreatic cancer [6], breast cancer [7,8], lung cancer [9], or medullary thyroid carcinoma (MTC). Moreover, some benign conditions can also present elevated CEA [10,11,12,13,14,15,16,17].

We present the case of persistent CEA elevation after complete remission of CRC and negative ^18^F-fluorodeoxyglucose (^18^F-FDG) PET/CT revealing localized MTC diagnoses with ^18^F-fluorodihydroxyphenylalanine (^18^F-FDOPA) PET/CT.

## 2. Case Report

A 70-year-old man was followed since 2014 with a pT2N1M0 left colon adenocarcinoma treated by left hemicolectomy then adjuvant chemotherapy (capecitabine and oxaliplatin). At the end of systemic treatments, carcinoembryonic antigen (CEA) was negative.

In 2015, CEA increased to 47 µg/L (N < 5) and ^18^F-fluorodeoxyglucose (^18^F-FDG) PET/CT showed a left adrenal hypermetabolism. The patient underwent a left adrenalectomy revealing a metastasis from the previous primary colon tumor, and post-operative CEA decreased to 5.6 µg/L. During follow-up, CEA gradually increased to 13.5 µg/L then stabilized around 12 µg/L. However, no lesions suspected of recurrence were found on several CT and ^18^F-FDG PET/CT.

**Figure 1:**
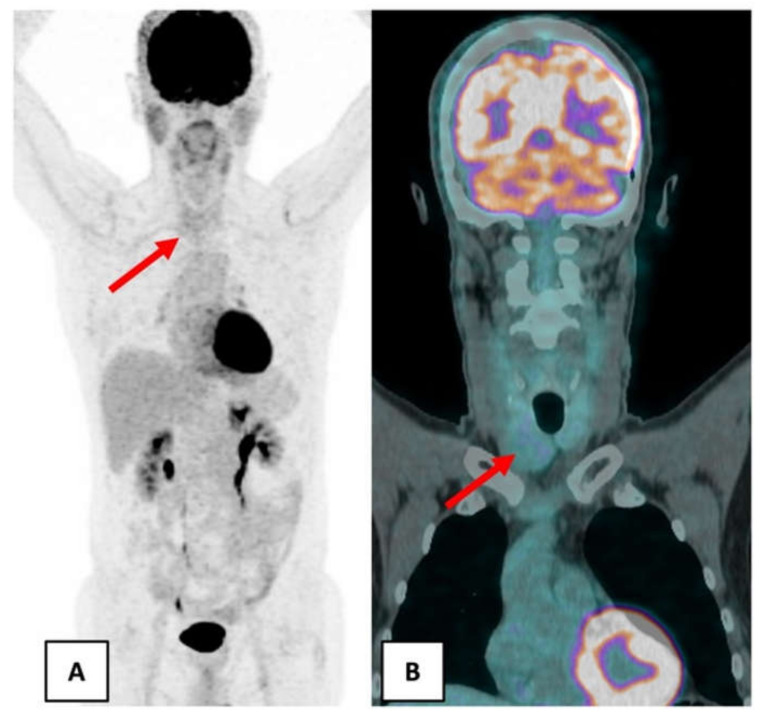
June 2018 ^18^F-FDG PET/CT showed a right lobar thyroid nodule without significant hypermetabolism (red arrow). (**A**) Maximum intensity projection. (**B**) Coronal fusion PET/CT.

In June 2018, as part of the follow-up, a new ^18^F-FDG PET/CT was performed in order to detect metastasis. The nuclear medicine physician noticed a right lobar thyroid nodule on the CT of ^18^F-FDG PET/CT without a significantly increased uptake (Figure 1).

In order to explore this nodule, cervical ultrasonography was performed and revealed a 28 × 22 × 36 mm hypoechoic nodule of the right lobe classified EU-TIRADS 5 (high risk of malignancy) [18]. Given the persistent elevation of CEA without secondary localization found and the presence of a thyroid nodule, an MTC was suspected.

Fine needle aspiration (FNA) was performed in this context of thyroid nodule with elevated calcitonin and CEA and found atypia of undetermined significance. Blood calcitonin was increased at 47 ng/L (N < 10). In our center, we do not routinely perform the calcium stimulated calcitonin test.

**Figure 2:**
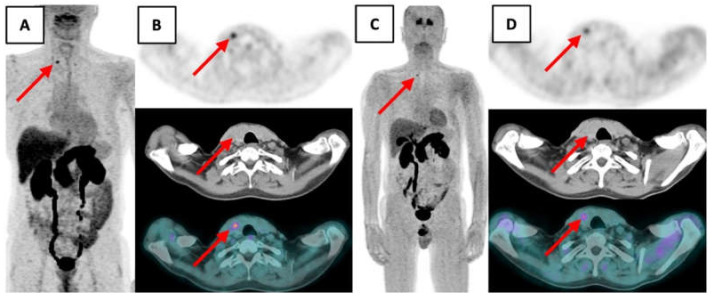
^18^F-FDOPA PET/CT showed a punctiform focus of the right thyroid lobe at the superior part of the nodular bulge suspected of MTC (red arrow).(**A**) Maximum intensity projection 12 min post-injection. (**B**) Transaxial PET, CT and fusion PET/CT 12 min post injection. (**C**) Maximum intensity projection 79 min post-injection. (**D**) Transaxial PET, CT and fusion PET/CT 79 min post injection.

Since our patient was reluctant to undergo surgery without any further exploration, we decided to perform a ^18^F-fluorodihydroxyphenylalanine (^18^F-FDOPA) PET/CT (Figure 2).

It showed a punctiform focus of the right thyroid lobe at the superior part of the visible nodular bulge with a maximum standardized uptake value (maxSUV) of 5.09 (12 min post-injection), and of 2.71 (79 min post-injection) compatible with a localized MTC.

A hemi-thyroidectomy with ipsilateral lymphadenectomy was performed in December 2018 because the patient wanted to avoid levothyroxine substitution. Histopathology confirmed a 1 cm MTC at the upper third of the right lobe without extrathyroidal extension nor vascular invasion, excised in the healthy zone and associated with a unique lymph node micrometastasis. It was classified pT1aN1aMxR0. One month after surgery, calcitonin was undetectable, and CEA decreased to the normal range at 2.6 µg/L. Genetic testing concluded a sporadic MTC (no *REarranged during Transfection (RET)* gene mutation).

## 3. Discussion

MTC is a rare neuroendocrine tumor derived from the C cells of the thyroid accounting for 1 to 2% of thyroid cancer in the United States. Although most MTC are sporadic, 25% are familial with germline mutations of the RET proto-oncogene resulting in multiple endocrine neoplasia (MEN) type 2A, type 2B, or familial medullary thyroid cancer (FMTC) with a phenotype-genotype correlation [19]. Sporadic MTC occurs in the fourth and sixth decades of life and is presented as a solitary nodule located in the upper portion of thyroid lobes [20]. At diagnosis, 70% of patients with a MTC presented as a palpable thyroid nodule have lymph node involvement and 10% have distant metastases.

There are two major prognostic factors at diagnosis: age and stage of the disease [19,21,22]. The 10-year specific survival is 81% in patients at any stage up to 96% in patient with localized MTC [23]. But microcarcinomas (≤1 cm) can also present distant metastases as shown by Kazaure et al. out of 310 patients with microcarcinomas, 5% had distant metastasis at diagnosis [24].

MTC cells are capable of secreting CEA and calcitonin used as a tumor marker. These two markers correlate with a tumor mass. Moreover, CEA and calcitonin doubling time are strong markers of the aggressiveness of MTC [25,26].

Despite the potential usefulness of ^18^F-FDG PET/CT in case of an unknown source of elevated CEA [27], this imaging may be falsely negative as in the case of a MTC, especially with a mild increase of serum calcitonin [28]. Therefore, after ruling out other non-malignant causes known to increase calcitonin such as renal failure, thyroiditis, sepsis, or other neuroendocrine tumors, an unexplained elevated CEA should lead to a calcitonin measurement in order not to miss a potential MTC [29].

Elevated CEA associated with elevated calcitonin is suggestive of MTC and further thyroid explorations are needed in particular cervical ultrasonography (US) to detect thyroid nodules. But physicians have to keep in mind that US risk stratification systems have been developed against papillary thyroid carcinoma and so there are no US features specifically for MTC [30]. In the same way, a FNA is usually performed to confirm the diagnosis of MTC despite its inconstant sensitivity, less than in other thyroid cancer, with a detection rate of only one-half of MTC [31]. This inconstant sensitivity could be increased by calcitonin measurement in the FNA needle washout fluids [32]. In case of mildly elevated CT, knowing MTC are difficult to diagnose with lack of sensitivity of US and cytology, a calcium stimulated calcitonin test could be performed [33].

In our case, ^18^F-FDG PET/CT was negative, and FNA cytology was non-contributory since it concluded atypia of undetermined significance, so we performed ^18^F-FDOPA PET/CT.

^18^F-FDOPA is a radiolabeled analog of DOPA that binds to an amino acid transporter (LAT1), which is overexpressed by neuroendocrine tumors, and which is converted to ^18^F-FDODA and stored in vesicles [34]. This imaging is currently the most sensitive and specific modality for detecting residual disease in patients with MTC and increased blood calcitonin levels [35,36], but there is no evidence of its performance to characterize a thyroid nodule.

As up to 6% of patients with sporadic MTC have bilateral foci [37], recommended treatment of localized MTC is total thyroidectomy and dissection of the lymph nodes in the central compartment [19]. Nevertheless, hemithyroidectomy with central neck dissection for sporadic MTC located in only one lobe did not affect the biochemical cure in selected patients [38].

So, given the absence of lymph node involvement shown by ultrasound and ^18^F-FDOPA PET/CT, blood calcitonin mildly elevated at 47 ng/L and the patient’s reluctance to purchases levothyroxine supplementation, hemithyroidectomy was an option.

## 4. Conclusions

Persistence of an increased CEA in case of colon adenocarcinoma without any secondary localization leading to the discovery of a MTC has already been reported [39,40,41] but for the first time, we report the contribution of ^18^F-FDOPA PET/CT in the pre-surgical characterization of a thyroid nodule. Moreover, this case demonstrates that a negative ^18^F-FDG PET/CT is not enough to exclude thyroid neoplasia, in particular, an indolent MTC and additional investigations are needed.

## Data Availability

The data presented in this study are available on request from the corresponding author.
